# On the Security and Privacy Challenges of Virtual Assistants

**DOI:** 10.3390/s21072312

**Published:** 2021-03-26

**Authors:** Tom Bolton, Tooska Dargahi, Sana Belguith, Mabrook S. Al-Rakhami, Ali Hassan Sodhro

**Affiliations:** 1 School of Science, Environment and Engineering, The University of Salford, Salford M5 4WT, UK; J.E.T.Bolton@salford.ac.uk (T.B.); t.dargahi@salford.ac.uk (T.D.); s.belguith@salford.ac.uk (S.B.); 2 Research Chair of Pervasive and Mobile Computing, Information Systems Department, College of Computer and Information Sciences, King Saud University, Riyadh 11543, Saudi Arabia; 3 Department of Computer and System Science, Mid Sweden University, SE-831 25 Östersund, Sweden; alihassan.sodhro@miun.se; 4 Shenzhen Institutes of Advanced Technology, Chinese Academy of Sciences, Shenzhen 518000, China; 5 Department of Electrical Engineering, Sukkur IBA University, Sukkur 65200, Pakistan

**Keywords:** virtual assistant, data security, privacy, GDPR, internet of things, smart homes

## Abstract

Since the purchase of Siri by Apple, and its release with the iPhone 4S in 2011, virtual assistants (VAs) have grown in number and popularity. The sophisticated natural language processing and speech recognition employed by VAs enables users to interact with them conversationally, almost as they would with another human. To service user voice requests, VAs transmit large amounts of data to their vendors; these data are processed and stored in the Cloud. The potential data security and privacy issues involved in this process provided the motivation to examine the current state of the art in VA research. In this study, we identify peer-reviewed literature that focuses on security and privacy concerns surrounding these assistants, including current trends in addressing how voice assistants are vulnerable to malicious attacks and worries that the VA is recording without the user’s knowledge or consent. The findings show that not only are these worries manifold, but there is a gap in the current state of the art, and no current literature reviews on the topic exist. This review sheds light on future research directions, such as providing solutions to perform voice authentication without an external device, and the compliance of VAs with privacy regulations.

## 1. Introduction

Within the last decade, there has been an increasing interest by governments and industry in developing smart homes. Houses are equipped with several internet-connected devices, such as smart meters, smart locks, and smart speakers to offer a range of services to improve quality of life. Virtual assistants (VAs)—often termed ‘smart speakers’—such as Amazon’s Alexa, Microsoft’s Cortana, and Apple’s Siri, simply described, are software applications that can interpret human speech as a question or instruction, perform tasks and respond using synthesised voices. These applications can run on personal computers, smartphones, tablets, and their dedicated hardware [1]. The user can interact with the VA in a natural and conversational manner: “Cortana, what is the weather forecast for Manchester tomorrow?”, “Alexa, set a reminder for the dentist”. The process requires no keyboards, microphones, or touchscreens [1]. This friction-free mode of operation is certainly gaining traction with users. In December 2017 there were 37 million smart speakers installed in the US alone; 12 months later this figure had risen to 66 million [2].

VAs and the companies behind them are not without their bad publicity. In 2018 the Guardian reported that an Alexa user from Portland, Oregon, asked Amazon to investigate when her device recorded a private conversation between her and her husband on the subject of hardwood floors and sent the audio to a contact in her address book—all without her knowing [3]. In 2019, the Daily Telegraph reported that Amazon employees were listening to Alexa users’ audio—including that which was recorded accidentally—at a rate of up to 1000 recordings per day [4]. As well as concerns about snooping by the VA, there are several privacy and security concerns around the information that VA companies store on their servers. The software application on the VA device is only a client—the bulk of the assistant’s work is done on a remote server, and every transaction and recording is kept by the VA company [5]. VAs have little in the way of voice authentication; they will respond to any voice that utters the wake word, meaning that one user could quite easily interrogate another’s VA to mine the stored personal information [1]. Additionally, Internet of Things (IoT) malware is becoming more common and more sophisticated [6]. There have been no reports yet of malware specifically targeting VAs ‘in the wild’ but it is surely a matter of time. A systematic review of research literature written on the security and privacy challenges of VAs and a critical analysis of these studies would give an insight into the current state of the art, and provide an understanding of any future directions new research might take.

### 1.1. Background

**Figure 1:**
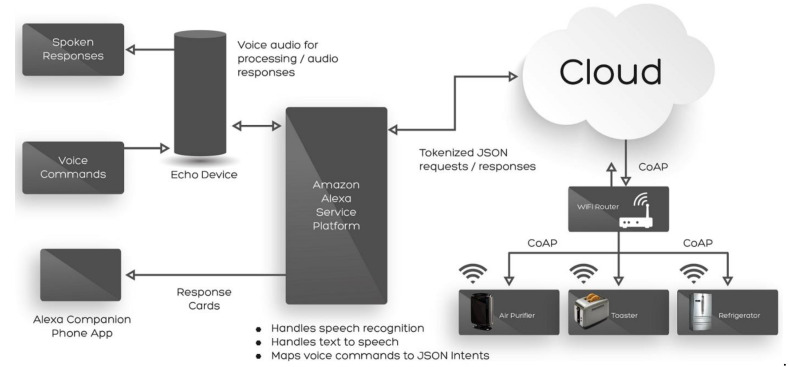
Architecture of a voice assistant (Alexa) (https://www.faststreamtech.com/blog/amazon-alexa-integrated-with-iot-ecosystem-service/). (access on 10 February 2021) [8].

The most popular VAs on the market are Apple’s Siri, Amazon’s Alexa, Microsoft’s Cortana, and Google’s Assistant [1]; these assistants, often found in portable devices such as smartphones or tablets, can each be considered a ‘speech-based natural user interface’ (NUI) [7]; a system that can be operated by a user via intuitive, natural behaviour, i.e., voice instructions. Detailed, accurate information about the exact system and software architecture of commercial VAs is hard to come by. Given the sales numbers involved, VA providers are perhaps keen to protect their intellectual property. Figure 1 shows a high-level overview of the system architecture of Amazon’s Alexa VA.

An example request might follow these steps:

1. The VA client—the ‘Echo Device’ in the diagram—is always listening for a spoken ‘wake word’; only when this is heard does any recording take place.
2. The recording of the user’s request is sent to Amazon’s service platform where the speech is turned into text by speech recognition, and natural language processing is used to translate that text into machine-readable instructions.
3. The recording and its text translation are sent to cloud storage, where they are kept.
4. The service platform generates a voice recording response which is played to the user via a loudspeaker in the VA client. The request might activate a ‘skill’—a software extension—to play music via streaming service Spotify, for example.
5. Further skills offer integration with IoT devices around the home; these can be controlled by messages sent from the service platform, via the Cloud.
6. A companion smartphone app can see responses sent by the service platform; some smartphones can also act like a fully-featured client.

As with any distributed computing system, there are several technologies used. The endpoint of the system with which the user interacts, shown here as the Echo device, commonly takes the form of a dedicated smart speaker—a computer-driven by a powerful 32-bit ARM Cortex CPU. In addition, these speakers support WiFi, Bluetooth, and have internal memory and storage [9].

The speech recognition, natural language processing (NLP), and storage of interactions are based in the Cloud. Amazon’s speech recognition and NLP service, known collectively as Amazon Voice Services (AVS) is hosted on their platform-as-a-service provider, Amazon Web Services (AWS). As well as AVS, AWS also hosts the cloud storage in which data records of voice interactions, along with their audio, are kept [10]. Data are transferred between the user endpoint and AVS using Javascript Object Notation-encoded messages via, in Amazon’s case, an unofficial public REST API hosted at http://pitangui.amazon.com (access on 22 February 2021) [11].

### 1.2. Prior Research and Contribution

There is a very limited number of systematic literature reviews (SLRs) written on the subject of VAs. To the best of our knowledge, none appears to specifically address the security and privacy challenges associated with VAs. The nearest that could be found was an SLR written by de Barcelos Silva et al. [12], in which a review of all literature pertinent to VAs is studied, and a relatively broad set of questions is posited and answered. Topics include a review of the state of the art, VA usage and architectures, and a taxonomy of VA classification. From the perspective of VA users who are motor or visually impaired, Siebra et al. [8] provided a literature review in 2018 that analysed VAs as a resource of accessibility for mobile devices. The authors identified and analysed proposals for VAs that better enable smartphone interaction for blind, motor-impaired, dyslexic, and other users who might need assistance. The end goal of their research was to develop a VA with suitable functions to aid these users. The study concluded that the current state of the art did not provide such research and outlined a preliminary protocol as a springboard for future work.

The main aim of this paper is to answer a specific question: “Are there privacy, security, or usage challenges with virtual assistants?” through a systematic literature review. A methodology was established for selecting studies made on the broader subject of VAs, and categorising them into more specific subgroups, i.e., subject audience, security or privacy challenges, and research theme (including user behaviour, applications, exploits, snooping, authentication, and forensics). In total, 20 papers were selected as primary studies to answer the research questions posited in the following section.

### 1.3. Research Goals

The purpose of this research was to take suitable existing studies, analyse their findings, and summarise the research undertaken into the security and privacy bearings of popular virtual assistants. Considering the lack of existing literature reviews on this subject, we aimed, in this paper, to fill the gap in the current research by linking together those studies which have addressed the privacy and security aspects of VAs in isolation, whether they have been written with users or developers in mind. To that end, the research questions listed in Table 1 have been considered.

The rest of this paper is organised as follows: the research methodology used to select the studies is outlined in Section 2, whereas Section 3 discusses the findings for the selection of studies, and categorises those papers. In Section 4, the research questions are answered, followed by a discussion on the future research directions in Section 5. Section 6 concludes the paper.

## 2. Research Methodology

In order to answer the research questions in Table 1, the following stages were undertaken.

### 2.1. Selection of Primary Studies

A search for a set of primary studies was undertaken by searching the website of particular publishers and using the Google Scholar search engine. The set of keywords used was designed to elicit results pertaining to security and privacy topics associated with popular digital assistants, such as Apple’s Siri, Google’s Assistant, and Amazon’s Alexa. To ensure that no papers were missed that might otherwise have been of interest, the search term was widened to use three further common terms for a virtual assistant. Boolean operators were limited to AND and OR. The searches were limited to the keywords, abstracts, and titles of the documents. The search term used was:

(“digital assistant” OR “virtual assistant” OR “virtual personal assistant” OR “siri” OR “google assistant” OR “alexa”) AND (“privacy” OR “security”)

Alongside Google Scholar, the following databases were searched:

- IEEE Xplore Library
- ScienceDirect
- ACM Digital Library

### 2.2. Inclusion and Exclusion Criteria

For a study to be included in this SLR, it must present empirical findings; these could be technical research on security or more qualitative work on privacy. The study could apply to end-users, application developers, or the emerging work on VA forensics. The outcome of the study must contain data relating to tangible, technical privacy, and/or security aspects of VAs. General legal and ethical studies, although interesting, were excluded. For a paper to be selected, it had to be fully peer-reviewed research; therefore, results that were taken from blogs, industry magazines, or individual studies were excluded. Table 2 outlines the exact criteria chosen.

### 2.3. Results Selection

Using the initial search criteria, 381 studies were singled out. These are broken down as follows:

- IEEE Xplore: 27
- ScienceDirect: 43
- ACM Digital Library: 117
- Google Scholar: 194

The inclusion and exclusion criteria (Table 2) were applied, and a checklist was assembled to assess the quality of each study:

- Does the study clearly show the purpose of the research?
- Does the study adequately describe the background of the research and place it in context?
- Does the study present a research methodology?
- Does the study show results?
- Does the study describe a conclusion, placing the results in context?
- Does the study recommend improvements or further works?

**Figure 2:**
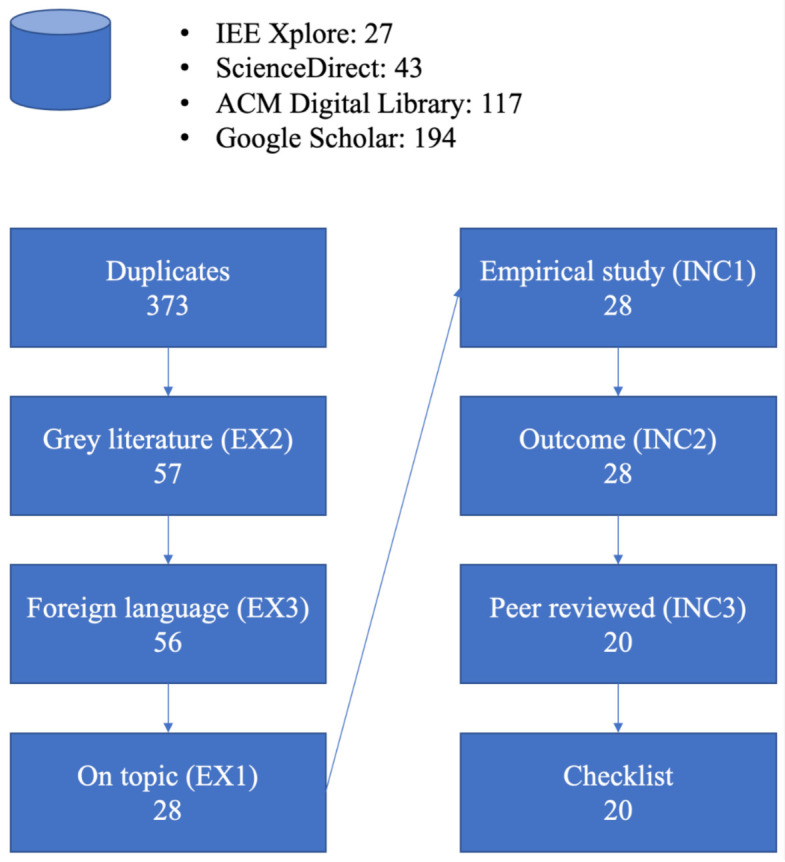
Attrition of papers at different processing stages.

EX2 (grey literature) removed 310 results, the bulk of the initial hits. Only one foreign-language paper was found amongst the results, which was also excluded. Throughout this process, eight duplicates were also found and excluded. With 63 results remaining for further study, these were read. A table was created using Excel and exclusion criterion EX1 (off-topic studies) was applied; following this, all three inclusion criteria were applied. Finally, 20 primary studies remained. Figure 2 shows how many studies remained after each stage of the process.

### 2.4. Publications over Time

**Figure 3:**
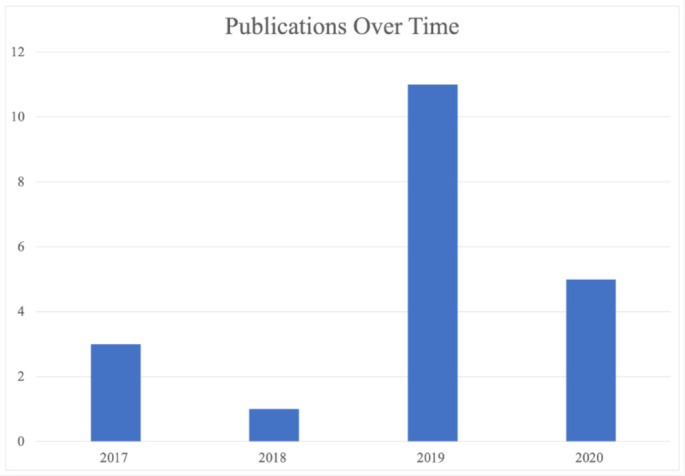
Number of primary studies against time.

If we consider the first popular VA to be Apple’s Siri [13]—first made available with the release of the company’s iPhone model 4S in 2011—it is interesting to see that the remaining primary studies which reported concrete data only dated back to 2017, four years before this review. The potential reasons for this will be discussed in Section 4. Figure 3 shows the number of publications by year.

## 3. Findings

From the initial searches, a large number of studies were found, perhaps surprisingly, given that VA technology is relatively young. It is only ten years since the introduction of the first popular VA, Apple’s Siri [13]. However, the attrition process described in Figure 2 reduced this number to 20.

**Figure 4:**
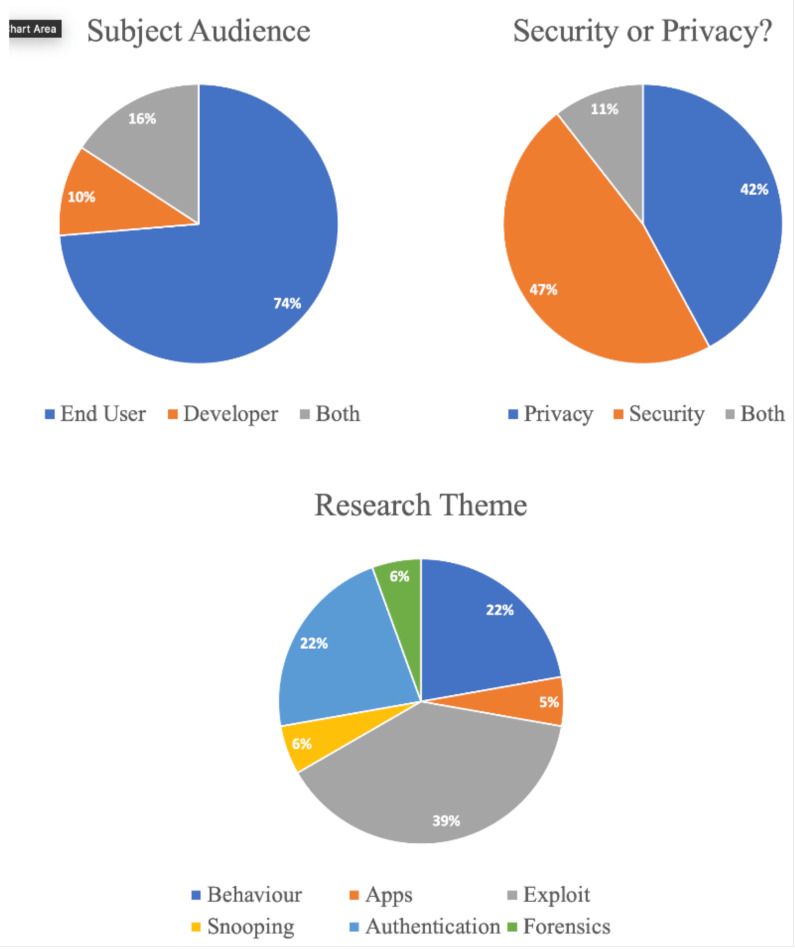
Visual representation of study classifications.

Instead of a single set of broad topics into which each of these studies could be categorised, we decided to approach each paper on three different levels, in line with the research questions posed in Section 1.3. The papers were divided into three categories: Subject Audience, Security and Privacy, and Research Theme. Figure 4 shows a visual representation of the breakdown of the individual categories.

### 3.1. Category 1: Subject Audience

The first categorisation is based on whether the work of the study is focussed on end-users, developers, or both.

End-users and developers are defined as follows:

- End-user—a person who uses the VA in everyday life. This person may not have the technical knowledge and may be thought of as a ‘customer’ of the company whose VA they have adopted.
- Developer—one who writes software extensions, known as ‘skills’ (Amazon) and ‘apps’ (Google). These extensions are made available to the end-user via online marketplaces.

### 3.2. Category 2: Security or Privacy?

As this study covers both security (safeguarding data) and privacy (safeguarding user identity), each study was categorised as one or the other. Only three papers covered both security and privacy in the same paper [14,15,16].

### 3.3. Category 3: Research Theme

The third categorisation considers the research themes addressed in each paper as follows:

- Behaviour—the reviewed study looks at how users perceive selected aspects of VAs, and factors influencing the adoption of VAs. All except one of the behavioural studies were carried out on a control group of users [11].
- Apps—the paper focuses on the development of software extensions and associated security implications.
- Exploit—the reviewed paper looks at malicious security attacks (hacking, malware) where a VA is the target of the threat actor.
- Snooping—the study is concerned with unauthorised listening, where the uninvited listening is being carried out by the device itself, as opposed to ‘Exploit’, where said listening is performed by a malicious threat actor.
- Authentication—the study looks at ways in which a user might authenticate to the device to ensure the VA knows whom it is interacting with.
- Forensics—the study looks at ways in which digital forensic artefacts can be retrieved from the device and its associated cloud services, for the purposes of a criminal investigation.

**Figure 5:**
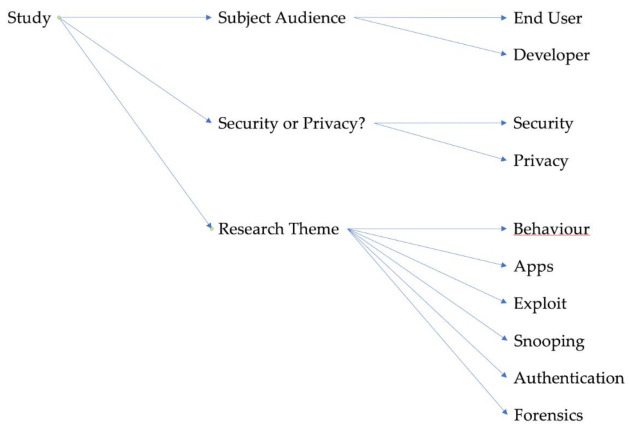
A taxonomy tree showing categories used to classify different reviewed papers.

A taxonomy tree showing these categories and how they relate to the studies to which they apply is shown in Figure 5.

It is worth noting that studies focusing on the theme of exploits—malware and hacking—were categorised as such if the VA was the target of the threat actor. Further classifying these studies’ audiences as end-users or developers also considers the nature of the exploit; both developers and end-users can be at risk from these attacks. When a malicious attack exploits a VA’s existing functionality, the study is categorised as ‘end-user’; it is the user who is affected by the exploit. Where the exploit requires new software to be written—for example, the creation of a malicious ‘Skill’—the study is categorised as both ‘developer’ and ‘end-user’ [10,17,18]. There was one study [19] that examined an exploit that required software to be written that exploited a vulnerability in other third-party software. Although the exploit may ultimately have affected the end-user, the focus there was on software development and so the paper was categorised as ‘developer’.

In terms of the subject audience, end-users were overwhelmingly the focus in 79% of papers; a further 11% included end-users with developers as the main focus, and 10% of papers were focussed only on developers. There was a fairly even split between security and privacy as the main thrust of the study; security was the subject of slightly more, at 47%, versus 42% for privacy. Few papers combined the study of both: only 11%. Examining the numbers in the research theme category, exploits were the focus of the majority of the studies; and behaviour was joint third alongside authentication as the focus of the remaining studies. The remainder—snooping, apps, and forensics—were split equally, with only one study dedicated to each. The primary studies are listed in Table 3, along with their categorisations.

## 4. Discussion

A recurring theme throughout this review so far has been the relative immaturity of VA technology and the short timeframe in which it has become widely adopted. There is, however, an interesting spread of subjects amongst the primary studies. Another interesting prevalence amongst the studies was that of the particular VA used as the subject of the research; of the papers that focused only on a particular VA, Amazon’s Alexa was the most popular as a subject.

In order to answer the research questions, each paper was read and the results were analysed. Each question is restated below, with a summary of key findings and a more in-depth precis of the studies to add context to the key findings.

### 4.1. RQ 1: What Are the Emerging Security and Privacy Concerns Surrounding the Use of VAs?

#### 4.1.1. Key Findings

While reviewing the papers, the following main findings were deduced:

- Successful malicious attacks have been demonstrated using VAs as the target [15,18,19,20,24]. These attacks are becoming more sophisticated, and some of them use remote vectors. These attacks are exploring different ideas, not just one vector.
- Personally identifiable information can be extracted from an unsecured VA with ease.
- The GDPR appears to be of limited help in safeguarding users in its current form.

#### 4.1.2. Discussion

From malicious attacks designed to impersonate a genuine skill or to bypass device authentication, to attacks designed to bypass VA device authentication, trends have emerged in both the security of VAs and the privacy of their users. Any attack that allows a malicious user to impersonate the user risks that user’s data falling into the wrong hands; attacks with a remote vector are of particular concern due to the comparative ease with which they could be launched without arousing the user’s suspicion. The cloud service platforms which power VAs store a lot of data and, should that data fall into the wrong hands, a serious privacy risk is exposed. The fact that two of the bigger vendors of VAs—Amazon and Google—have skill stores which allow the uploading of malicious applications deliberately designed to access a user’s data means that the user is unable to rely on the fact that the skill they downloaded and use is safe—a serious security concern.

The dolphin attack, as demonstrated by Zhang et al. [24], shows how Alexa can be manipulated by voice commands that are modulated to frequencies beyond the upper range of human hearing—an attack that requires planning, sophisticated equipment, and physical proximity to the VA device and therefore realistically poses a limited threat to the user. Turner et al. [18] showed that phoneme morphing could use audio of a source voice and transform it into an audio utterance that could unlock a device that used voice authentication. The original recording need not be that of the device user, which presents a security risk, but one that still relies on physical access to the VA device.

A man-in-the-middle attack called Lyexa was demonstrated in [19] by Mitev et al., in which a remote attacker uses a compromised IoT device in the user’s home, capable of emitting ultrasound signals, to ‘talk’ to the user’s VA. To further develop this idea from the dolphin attack [24], a malicious Alexa skill was used in tandem to both provide plausible feedback to the user from the VA to prevent the arousal of suspicion, and make this attack remote, thus increasing its threat potential. Kumar et al. [15] demonstrated a skill attack that is predicated on Alexa misinterpreting speech. It was shown that Alexa, in testing, correctly interpreted 68.9% of 572,319 words; 24 of these words were misinterpreted consistently, and when used by a malicious skill could be used to confuse genuine skills, thus providing a reliable, repeatable remote attack vector. In [27], Kennedy et al. demonstrated a particularly advanced form of an exploit that uses machine learning to derive patterns or ‘fingerprints’ and compares them with encrypted traffic between the VA and the server. Certain voice commands could be inferred from the encrypted traffic. This attack is a remote attack and consequently poses a serious security concern.

In conclusion, it was found that the VA is becoming the target of malicious attacks just as other connected computing devices have been in the past. These attacks show an interesting pattern: they are evolving. For any malicious attack to be effective and dangerous to the end user, it must be simple enough to be carried out by someone who has not made an extensive study of the VA’s internal architecture. Furthermore, an attack is made more dangerous by the lack of the need to be proximate to the device. Finally, any attack must be repeatable—if it only works once, in laboratory conditions for example, it poses little threat to the end user. A ready-coded, malicious skill could be exploited remotely by a threat actor with limited knowledge of computer science and it surely, at this point, cannot be long before these attacks are more commonplace.

Furey et al. [22] studied firstly how much personally identifiable information could be extracted from an Alexa device that had no authentication set. The authors then examined this in the context of GDPR, and how much leeway Amazon might have to offload their compliance responsibilities with carefully written user terms and conditions. Loideain et al. investigated how the female gendering of VAs might pose societal harm “insofar as they reproduce normative assumptions about the role of women as submissive and secondary to men” [26]. In both cases, the GDPR as it currently stands was found to be only partially successful in protecting VA users. The GDPR, designed expressly to protect the end user and their data, has been shown by two studies in this group to be of limited utility. A study of the GDPR itself or an analysis of the psychological repercussions of VA voice gendering are beyond the scope of this document. However, any flaws in GDPR are a particular concern, given the amount of data collected by VAs, and the increase in interest in exploiting vulnerabilities in VAs and their extensions in order to obtain these data by nefarious means.

### 4.2. RQ2: To What Degree Do Users’ Concerns Surrounding the Privacy and Security Aspects of VAs Affect Their Choice of VA and Their Behaviour around the Device?

#### 4.2.1. Key Findings

The review of the selected papers led to the following main findings:

- Rationalising of security and privacy concerns is more prevalent among those who choose to use a VA; those who don’t use one cite privacy and trust issues as factors affecting their decision.
- Conversely, amongst those who do choose to use a VA, privacy is the main factor in the acceptance of a particular model.
- ‘Unwanted’ recordings—those made by the VA without the user uttering the wake word—occur in significant numbers.
- Children see no difference between a connected toy and a VA designed for adult use.

#### 4.2.2. Discussion

Lau et al. [17] found that worries differ between people who do and do not use a VA. Those who do not use an assistant, refusing to see the purpose of such a device, are more likely to be the subjects for whom privacy and trust are an issue. These users were “…deeply uncomfortable with the idea of a ‘microphone-based’ device that a speaker company, or an ‘other’ with malicious intent, could ostensibly use to listen in on their homes”. Amongst those who do adopt a VA, users rationalised their lack of concern regarding privacy with the belief that the VA company could be trusted with their data, or that there was no way another user could see their history. Burbach et al. considered the acceptance factors of different VAs amongst a control group of users; a choice-based conjoint analysis was used, having three attributes: natural language processing (NLP) performance, price, and privacy. Privacy was found to be the biggest concern of the three [14]. These findings appear to conflict with those presented by Lau et al. [21]; however, the construction of the surveys was different, as privacy was the primary goal of the study. Moreover, Burbach et al. [11] wrote their study a year later; a year in which several news stories broke in the media regarding privacy concerns of VAs, which may account for the apparent increase in concern over privacy.

Javed et al. [21] performed an in-depth study of what Alexa was recording. AlthoughAmazon claims that ‘she’ only listens when the wake-word is uttered by the user, their research found that among the control group of users, 91% had experienced an unwanted recording. This was investigated and it was found that benign sounds such as radio and TV and background noise, were recorded in the majority of these cases. Alarmingly, however, 29.2% of the study group reported that some of their unwanted recordings contained sensitive information, which presents a privacy breach. McReynolds et al. studied connected toys (Hello Barbie, Jibo) in conjunction with VAs to determine, amongst other questions, if children relate to ‘traditional’ smart assistants in the same way they do their toys [29]. A key finding was that having surveyed groups of parents and their children, VAs were used by children who interacted with them in the same way they might interact with a connected toy. VAs, however, are not designed for children and are not examined—at least in the US—for regulatory compliance in the same way connected toys are.

Although there has been an increase in user privacy concerns, there is still a group of users who have faith that the data companies are trustworthy; interestingly, a group of those users for whom privacy is a concern are still using a VA. The fact that privacy is a worry is evidently not sufficient to dissuade the user from having a VA in the house. It might be interesting to see if studies made over the coming years show the trend of privacy awareness continuing, especially in the light of the simple fact that users find VAs recording without their knowledge. Children relate to VAs as they would a toy with similar capabilities and, again, it would be of interest to see if this fact increased privacy concerns amongst parents who use an ‘adult’ VA.

### 4.3. RQ3: What Are the Security and Privacy Concerns Affecting First-Party and Third-Party Application Development for VA Software?

#### 4.3.1. Key Findings

The study of the selected papers led us to deduce the following main findings:

- The processes that check third-party extensions submitted to the app stores of both Amazon and Google do a demonstrably poor job of ensuring that the apps properly authenticate from the third-party server to the Alexa/Google cloud.
- Several novel methods of user authentication to the VA device have been proposed, each using a different secondary device to offer a form of two-factor authentication [16,23,31].
- Each of the user authentication methods do go some way to mitigating the voice/replay attacks outlined in the findings of RQ1.

#### 4.3.2. Discussion

Zhang et al. [14] presented the only study which examined security vetting processes used by the VA manufacturers; these procedures are put in place to ensure that developers of third-party VA extensions (‘skills’, ‘apps’) are ensuring that proper security is implemented in their code. As their research demonstrates, vulnerable extensions—voice squatting attacks, written by the authors to specifically target a genuine skill—have been approved by both Amazon and Google. Combined with the findings in RQ1, in which several VA attacks were identified that relied on malicious extensions, this finding represents a significant security risk. The authors went so far as to inform both Amazon and Google of their findings and have consequently met with both companies in order to help the organisations better understand the novel security risks.

Moving away from extension application development, three novel approaches that might suggest a better way in which VA companies might improve security for end-users have been proposed. Feng et al. [23] presented what they call ‘VAuth’, a method of ‘continuous’ authentication, in which a wearable device collects unique body surface vibrations emanating from the user and matches them with the voice signal heard by the VA. Wang et al. [31] proposed another wearable that might provide two-factor authentication. In this approach, termed ‘WearID’, however, the wearable in this instance captures unique vibration patterns not from the user’s body but from the vibration domain of the user’s voice. These are then used in tandem with existing device authentication.

Cheng et al. [16] suggested ‘acoustic tagging’, whereby a secondary device emits a unique acoustic signal, or ‘watermark’, which is heard in tandem with the user’s voice. The VA—registered to the user—may then accept or reject voice audio instructions accordingly. All three of these methods of authentication go some way towards mitigating malicious attacks, such as the dolphin attack demonstrated by Zhang et al. [24]. They also provide an extra layer of security for those users concerned about privacy by making it much harder for another user to access a VA without permission. However, they can be considered a form of two-factor authentication, as each of the studies proposes a method that requires extra hardware. Two studies [23,31] involved the use of wearables which might not always be practical for multiple users, as well as adding extra expense and complication for the user.

To conclude, there are worrying security considerations around VAs. Methods of two-factor authentication with an external device, although sophisticated, are cumbersome for users. Interestingly, there were no works at the time of our study on authenticating a user entirely based on their voice fingerprint. Given the lack of vetting in the major vendors’ application stores, which is itself a vulnerability open to exploitation, securing the VA is absolutely essential.

## 5. Open Research Challenges and Future Directions

According to the results of this study, it can be seen that VAs, like any other computing device, are vulnerable to malicious attacks. A number of vulnerabilities have been studied, and several attacks have been crafted that take advantage of flaws in the design of the VA itself and its software extensions. It has also been shown that VAs can mishear their wake words and make recordings without the user’s knowledge and, even when the user is aware, the VA vendor is recording and storing a large amount of personal information. Therefore, the security and privacy of VAs are still challenging and require further investigation. Three main future research directions are identified and discussed in the following sections.

### 5.1. GDPR and the Extent of Its Protections

Although an increase in users’ privacy awareness can be seen, among significant numbers of users there is still an alarming—almost blind—reliance on vendors such as Amazon and Google to ‘do the right thing’ and treat the user’s data responsibly and fairly in accordance with GDPR or other local data regulations. Future work might examine whether or not the vendors are fully complying with data law or whether they are adhering to it as little as possible in order to make their businesses more profitable. The work might also study whether or not regulations, such as GDPR, are offering as much protection to the end-user as they should and, if not, where they are failing and need improvement.

### 5.2. Forensics

Although studies on the forensic aspects of VAs have to date concentrated on finding as much information as possible both from the device and the cloud service platform, little work appears to have been carried out on examining exactly what is stored. Future work could look at how VAs interact with their cloud service providers, and how open the interfaces between the device and server are. Furthermore, it is not clear how much the user is (or can be) aware of what is being stored. This presents an interesting imbalance; while it is possible for the user to see certain data that are stored, the vendors’ ‘privacy dashboards’ through which this information can be gleaned are not telling the whole story. Future work might study this imbalance and find ways in which the user might become more aware of the extent of the data that are being taken from them, stored, and manipulated for the vendors’ profit.

### 5.3. Voice Authentication without External Device

As discussed in this paper, VA user authentication is a concern, as with any other service that collects user data. A VA collects substantial amounts of personal data, as demonstrated in the forensics-focussed works studied in this paper. Several novel methods for authenticating a user to their device were presented in the primary studies. However, each used an external device to provide a form of two-factor authentication, which makes the resultant solution cumbersome and complicated for the user. An interesting future research direction could address this challenge by focusing on biometric voice analysis as a means of authenticating the user, rather than relying on an external device.

## 6. Conclusions

In this paper, based on a systematic literature review on the security and privacy challenges of virtual assistants, several gaps in the current research landscape were identified. Research has been carried out on the themes of user concerns, the threat of malicious attack, and improving authentication. However, these studies do not take an overarching view of how these themes may interact, leading to a potential disconnect between these areas. A number of studies concentrated on user behaviour, identifying privacy and security concerns; however, they did not mention how these concerns might be addressed, except [33], in which a few suggestions were provided for privacy and security design, including improvements to muting, privacy default settings, and audio log features, as well as adding security layers to voice recognition and providing offline capabilities. In addition, it was found that when one particular VA was the focus of the study, Amazon’s Alexa was the assistant that was chosen in the majority of these papers. Given Amazon’s sales dominance in the smart speaker sector, this is perhaps understandable. There are, however, many more VA systems that might be going uninvestigated as a consequence.

The results from answering research question 1 in this study showed that increasingly sophisticated malicious attacks on VAs are being demonstrated, and yet user awareness of this specific and worrying trend appears not to have been studied in any great detail. The three research questions posited were answered as follows. (1) There were several emerging security and privacy concerns, (2) security and privacy concerns do affect users’ adoption of VAs and adoption of a particular model of VA, and (3) there are worrying concerns and security lapses in the way third party software is vetted by manufacturers. It would be interesting to investigate further how these areas converge, as the current research, although it is of great use in its own subject area, can have a narrow focus. It would be fascinating if knock-on effects to other areas could be further researched by broadening the focus areas investigated.

## Figures and Tables

**Table 1:** Research questions.

Research Question	Discussion
**RQ1:** What are the emerging security and privacy concerns surrounding the use of VAs?	Virtual assistants have become more and more commonplace; as a consequence, the amount of data associated with their use and stored by the VA companies will have commensurately increased [2]. A review of current research will help to understand exactly how private and secure these data are from a user’s perspective. As well as this, we will better understand what risks there are and how they can, if possible, be mitigated.
**RQ2:** To what degree do users’ concerns surrounding the privacy and security aspects of VAs affect their choice of VA and their behaviour around the device?	As consumers adopt more technology, do they become more aware of the security and privacy aspects around the storage of these data? In the current climate, ‘big data’ is frequently in the news, and not always in a positive light [3,4]. Do privacy and security worries affect users’ decisions to select a particular device more than the factor of price, for instance, and do these worries alter their behaviour when using the device? Reviewing current research will give us empirical data to answer this question.
**RQ3:** What are the security and privacy concerns affecting first-party and third-party application development for VA software?	A review of research into how the development of VA software and its extensions is changing will highlight the privacy and security concerns with regard to these extensions, and how developers and manufacturers are ensuring that they are addressed. Additional insights might come from those in the research community proposing novel ideas.

**Table 2:** Inclusion and exclusion criteria for study selection.

Criteria for Inclusion	Criteria for Exclusion
INC1: The paper must present an empirical study of either security or privacy aspects of digital assistants.	EX1: Studies focusing on topics other than security or privacy aspects of digital assistants, such as broader ethical concerns or usage studies. These studies might have a passing interest in security or privacy, but not focus on these as the main investigation.
INC2: The outcome of the study must contain information relating to tangible privacy or security elements.	EX2: Grey literature—blogs, government documents, comment articles.
INC3: The paper must be full research, peer reviewed, and published in a journal or conference proceedings.	EX3: Papers not written in English.

**Table 3:** Key data reported by primary studies.

Research Paper	Key Findings	Categories
Burbach et al. [11]	This paper studied user acceptance of particular VAs, and the factors influencing the decision to adopt one over the other. The relative importance of language performance, price, and privacy were observed among a control group of participants. The authors devised a choice-based conjoint analysis to examine how each of these attributes might affect the acceptance or rejection of a VA. The analysis took the form of a survey divided into three parts—user-related factors (age, gender), participants’ previous experience with VAs (in the form of a Likert scale), and self-efficacy (the users’ ability to operate the technology). The results found a fairly representative female–male split (53% to 47%) in terms of who tended towards an affinity with technology. Of particular interest was one question asked of the participants—“how would you react if someone were to install a VA without asking”—at which most of the participants protested.	1. End-User 2. Privacy 3. Behaviour
Zhang et al. [14]	A case study of voice masquerading and voice squatting attacks using malicious skills; in this paper, the authors were able to successfully circumvent security vetting mechanisms used by Amazon and Google for checking submissions of apps written by third-party extension developers. The paper demonstrated that malicious applications can pass vetting, and the authors suggested novel techniques for mitigating this loophole. The authors have subsequently approached both Amazon and Google with their findings and have offered advice on how such voice squatting attacks via malicious skills might be prevented from entering their app stores.	1. Developer 2. Security 3. Apps
Castell-Uroz et al. [20]	This paper identified and exploited a potential flaw in Amazon’s Alexa which allows the remote execution of voice commands. The authors first analysed network traffic to and from an Echo Dot smart speaker using man-in-the-middle techniques in Burp Suite; however, the privacy of the communications was deemed to be sufficiently robust. The flaw was uncovered using an audio database of some 1700 Spanish words played near the device. Using those words which were able to confuse the device into waking, in combination with a voice command as part of a reminder, the device was found to ‘listen’ to itself and not discard the command. The attack, although not developed further, was deemed by the authors to be sufficient for a malicious user to make online purchases with another user’s VA.	1. End-User/Developer 2. Security 3. Exploit
Mitev et al. [19]	This paper demonstrated a man-in-the-middle attack on Alexa using a combination of existing skills and new, malicious skills (third-party extensions). It showed more powerful attacks than those previously thought possible. The authors found that skill functionality can be abused in combination with known inaudible (ultrasound) attack techniques to circumvent Alexa’s skill interaction model and allow a malicious attacker to “arbitrarily control and manipulate interactions between the user and other benign skills.” The final result was able to hijack a conversation between a user and VA and was very hard to detect by the user. The new-found power of the attack stemmed from the fact that it worked in the context of active user interaction, i.e., while the user is talking to the device, thus maintaining the conversation from the user’s perspective. Instead of launching simple pre-prepared commands, the attack was able to actively manipulate the user.	1. Developer 2. Security 3. Exploit
Lau et al. [17]	A study demonstrating end-user VA behaviour, along with users’ privacy perceptions and concerns. The paper presented a qualitative analysis based on a diary study and structured interviews. The diary study took the form of semi-structured interviews with 17 users and, for balance, 17 non-users of VAs. Users were asked to diarise instances of using the device and of accidental wake-word triggerings at least once per day for a week. This was followed up by an interview in the homes of the users, taking into account details such as where the device was placed and why. Non-users were interviewed separately and asked questions pertaining to their choice to not use a VA and privacy implications that might have had a bearing in the choice. Qualitative analysis of the interviews used a derived codebook to analyse and identify running themes and emergent categories. Results identified who was setting up the speaker (the user or another person), speaker usage patterns, and placement of the speaker according to perceived privacy of certain house rooms.	1. End-User 2. Privacy 3. Behaviour
Javed et al. [21]	A study based on the hypotheses that Alexa is recording conversations without being woken, users are not aware of this, and it is a concern of users. In this paper, a study was made of Alexa’s recording, a survey of users was undertaken, and quantitative analysis performed. Participants in the survey were first screened to ensure they owned an Alexa-enabled device; records were made of the participants’ demographics. The main part of the survey asked participants about the VA’s data storage, access, and deletion behaviour; questions asked of the participants were also designed to demonstrate their awareness and perceptions of the VA’s unintended voice recording. Finally, the participants were asked questions according to Westin’s privacy classification to determine their level of concern about privacy, which were then used to make a quantitative analysis. Further results were obtained by forming three hypotheses about users’ perceptions in certain areas to see how many participants have correct or incorrect ideas about how Alexa works.	1. End-User 2. Privacy/Security 3. Snooping
Turner et al. [18]	This paper presented a demonstration of a security attack, ‘phoneme morphing’, in which a VA is tricked into thinking a modified recording of an attacker’s voice is the device’s registered user, thus fooling authentication. It demonstrated the attack and quantitively analysed the variance in several attack parameters. The attack was predicated on a method which mapped phenomes (of which there are 44 in the English language) uttered by a known speaker, into phenomes resembling those spoken by the victim. Three stages of the attack were determined: offline, the phenome clustering of the source voice was performed; a recording of the victim’s voice was obtained to map phenomes between the source and target; finally, the transformed audio was played to the system. The attack’s success was measured using four key phrases, with significant variance in success—the lowest being 42.1% successful, and the highest being 89.5% effective.	1. End-User 2. Security 3. Exploit
Furey et al. [22]	The paper examined the relationship between trust, privacy, and device functionality and the extent to which personally identifiable information (PII) was retrievable by an unauthorized individual via voice interaction with the device. The authors made a qualitative analysis of privacy breaches, and the extent to which General Data Protection Regulation (GDPR) has helped to address these. Using a script of voice queries to be asked of the target device (an Amazon Echo Dot), an unauthorized user was granted a five-minute session with the speaker to determine which of the script’s questions could extract PII from the device. The device itself was linked to several other accessories—a smartphone and a fitness watch—and the questions asked corresponded with GDPR’s definition of what may constitute PII. Results of PII were tabulated, as a demonstration of such information (sleep location, contacts, schedule) could be obtained.	1. End-User 2. Privacy 3. Exploit
Feng et al. [23]	The study proposed and demonstrated a continuous authentication model through the use of wearables. Quantitative analysis of the demonstration’s results, when compared with existing methods, was presented. The authors’ proposed solution, ‘VAuth’, continuously samples the user’s speech to ensure that commands originate from the user’s throat. VAuth, shown as a prototype device attached to a pair of spectacles, connects to the VA via Bluetooth and performs the speech comparison using an extension app, or skill. By using a skill, the comparison code can make use of server-side computing power. Built using Google Now in an Android host, VAuth was tested on 18 participants who each uttered 30 separate voice commands. The system detected the users with an overall 97% accuracy rate.	1. End-User/Developer 2. Security 3. Authentication
Zhang et al. [24]	The paper demonstrated the ‘dolphin attack’, in which inaudible (freq. > 20 KHz) voice commands can be used to communicate with VAs, evading detection by human hearing. The paper presented a quantitative analysis of various attack parameters, and performed a successful demonstration on a number of Vas, including Siri, Google Now, Samsung S Voice, Cortana, and Alexa. Results of the experiment were ultimately tabulated, showing the device used, whether the command was recognized by the device, and then whether the command resulted in device activation. The maximum distances from the VA device’s microphone were also recorded—an important point of note, as the maximum distance was 1650 mm, indicating that the attack, although successful, does rely on being proximate to the device in question.	1. End-User 2. Security 3. Exploit
Kumar et al. [15]	This paper was a demonstration of ‘skill squatting’ attacks on Alexa, in which a malicious skill is used to exploit the VA’s misinterpretation of speech to perform complex phishing attacks. Kumar et al. presented a successful demonstration and quantitative analysis and classification of misinterpretation rates and parameters, and highlighted the potential for exploitation. The attack was predicated on the use of speech misinterpretations to redirect a user towards a malicious skill without their knowledge. The authors first used a speech corpus to provide structured speech data from a range of American subjects. Finding that Alexa only managed to correctly interpret 68.9% of the words in the corpus, the authors were able to classify interpretation errors (homophone, phonetic confusion). They were then able to identify existing genuine skills with easily confusable names (“cat facts” becomes “cat fax”) and use predictable errors to redirect users to a malicious skill of their construction. As a counterpoint, the authors offered some measures which Amazon et al. might take to prevent such malicious skills from entering the app store, including phoneme-based analysis of a new skill’s name during the store’s certification process.	1. End-User/Developer 2. Security 3. Exploit
Yıldırım et al. [25]	This study presented an overview of Amazon and Google VAs as a source of digital forensic evidence. A brief study was undertaken with qualitative analysis. The study was predicated on searching a VA device (a Samsung smartphone) for activity history entries relating to voice commands that had been issued. Data including the voice command in text form, timestamps, and the assistant’s response were found. No study was made of the cloud service platforms used to power the VAs in question; only the local device was examined.	1. End-User 2. Privacy 3. Forensics
Loideain et al. [26]	Loideain et al. presented a qualitative study on the gendering of VAs, and the consequential societal harm which might result. VAs are generally gendered decisively as female and the authors argued that this gendering may enforce normative assumptions that women are submissive and secondary to men. The paper examines how GDPR and other data protection regulations could be used to address the issue; in particular, the study branched out into asking questions about the further role that data regulation might take in in AI design choices in areas such as equality and consumer law.	1. End-User 2. Privacy 3. Behaviour
Kennedy et al. [27]	This paper studied a fingerprinting attack in which an eavesdropper with access to encrypted VA network traffic can use the data to correctly infer voice commands using machine-learning-derived fingerprints. An in-depth quantitative analysis of the attack metrics and success rate was introduced. The programming language Python was used to process traffic obtained from the network using the tool Wireshark; over 1000 network traces were obtained. The author’s software, ‘eavesdroppercollecting’, inferred voice commands from encrypted traffic with 33.8% accuracy. The authors went on to address the limitation in similar attacks which adopt this accuracy as their only metric; they proposed ‘semantic distancing’ as a further metric, whereby an attacker might infer a voice command that was different from, yet similar to, the original command.	1. End-User 2. Privacy 3. Exploit
Sangal et al. [28]	A study of safety issues surrounding the use of VAs by children. The paper offered a qualitative analysis of the problem and a proposal for a solution, with an analysis of the success rate thereof. The proposed solution aimed to address the problem that a VA, in normal circumstances and with no authentication enabled, can be used by anyone in its vicinity, whether child or adult. Several AI algorithms were posited, based on such metrics as voice frequency (assumed to be higher in a child), intended to form part of an improved service by the VA provider. Google and Amazon were used in the study.	1. End-User 2. Security/Privacy 3. Authentication
Cheng et al. [16]	The paper proposed a novel method of ‘watermarking’ user interaction with a VA (Google Home). The authors presented a quantitative and qualitative analysis of the problem and the success of the proposed solution. The proposed solution took the form of an acoustic ‘tag’—a small, wearable device that emits an audible signal unique to that tag which can act as a form of authentication that is far more sophisticated than a standard VA wake word. In this instance, the authentication is not continuous—that is, it is only used at the start of a transaction, similar to any PIN or password. The authors experimented with tags that emitted audible, unnoticeable, or hidden signals. An analysis of the chosen design implementation (an audible tag) was carried out using a Google Home smart speaker, of which the audio capabilities (recording fidelity and sampling rates) were known beforehand.	1. End-User 2. Security 3. Authentication
McReynolds et al. [29]	The paper presented a study of privacy perceptions surrounding ‘connected toys’. It introduced a quantitative analysis of data gathered through interviews and observation. Two connected toys were used, ‘Hello Barbie’ and ‘CogniToys Dino’. Semi-structured interviews with parent-child pairs were conducted, covering three research questions—general interaction, privacy, and parental controls. Child participants were aged between six and 10 years. While watching the child play with the toys, the parents were asked the first set of questions. The second and third sets of questions were asked after the parent and child had been separated. The interviews were transcribed, and a codebook was developed to categorise the responses to the interview questions.	1. End-User 2. Privacy 3. Behaviour
Wei Li et al. [30]	The authors proposed a novel way of ‘encrypting’ user voice commands using the granule computing technique. The paper detailed a quantitative analysis of the problem and the proposal’s success. Unlike existing VA client endpoints—of which the computing is used primarily to listen for a wake word and to sample subsequent audio information for transportation to the cloud for processing—the author’s model performed most of the computing on the VA device. Each sound could be encrypted using the advanced encryption standard (AES), using a different key for each voice, decreasing the likelihood that a malicious attacker could decrypt the content.	1. End-User 2. Privacy 3. Authentication
Wang et al. [31]	Wang et al. proposed ‘WearID’, whereby a smartwatch or other wearable is used as a secure token as a form of two-factor authentication to a VA. The paper presented a quantitative analysis of the proposal’s success rate and an in-depth analysis of the problem. WearID uses motion sensors—accelerometers—to detect the airborne vibrations from the user’s speech and compares it to known values using cross-domain analysis (sampled audio vs. vibration) to authenticate the user. The authors proposed that the technology could be used “under high-security-level scenarios (e.g., nuclear power stations, stock exchanges, and data centers), where all voice commands are critical and desire around-the-clock authentication.” WearID was shown, using two prototype devices (smartwatches) and 1000 voice commands, to correctly authenticate users with 99.8% accuracy, and detect 97.2% of malicious voice commands, both audible and inaudible.	1. End-User 2. Security 3. Authentication
Chalhoub and Flechais [32]	The paper presented a study on the effect of user experience on security and privacy considerations of smart speakers. It introduced qualitative and quantitative analysis of data gathered through theoretical reasoning and interviews. The authors discovered factors influencing smart speaker adoption and security/privacy perception and trade-offs between the two. Interviews were coded with grounded theory for analysis; 13 participants were involved in the study. The themes of the interview included perceptions and beliefs towards privacy resignation, the usability of security controls, trigger points for security and privacy considerations, factors for adoption, and privacy/security tradeoffs with User Experience (UX) personalization. The study found that users reported ‘compensatory behaviour’ towards non-user-friendly security and privacy features.	1. End-User 2. Security and Privacy 3. Behaviour
